# The Role of HLA-Class Ib Molecules in Immune-Related Diseases, Tumors, and Infections

**DOI:** 10.1155/2014/231618

**Published:** 2014-05-28

**Authors:** Fabio Morandi, Enrico Fainardi, Roberta Rizzo, Nathalie Rouas-Freiss

**Affiliations:** ^1^Laboratory of Oncology, Istituto Giannina Gaslini, Via Gerolamo Gaslini, No. 5, 16147 Genova, Italy; ^2^Neuroradiology Unit, Department of Neurosciences and Rehabilitation, Azienda Ospedaliera-Universitaria, Arcispedale S. Anna, Via Aldo Moro, No. 8, Cona, 44124 Ferrara, Italy; ^3^Department of Experimental and Diagnostic Medicine, Section of Microbiology, University of Ferrara, Via Savonarola, No. 9, 44121 Ferrara, Italy; ^4^CEA, Institut des Maladies Émergentes et des Thérapies Innovantes (iMETI), Service de Recherche en Hémato-Immunologie (SRHI), Hôpital Saint-Louis, Avenue Claude Vellefaux 1, 75010 Paris, France

HLA-class Ib family include HLA-E, -F, -G, and -H molecules that, in contrast with high polymorphic HLA-class Ia molecules (HLA-A, -B, and -C), display a limited polymorphism, with a small number of alleles encoding limited functional proteins. Similar to HLA-class Ia molecules, HLA-class Ib molecules bind peptides generated from cytosolic antigens and present them to CD8^+^ T lymphocytes, but their main function is the regulation of immune responses, both in physiological and pathological conditions.

HLA-G, the best characterized HLA-Ib molecule, is expressed on fetal cytotrophoblast cells during pregnancy and abrogates maternal NK cell cytotoxicity against fetal tissues. However, HLA-G is also expressed (or released as soluble molecules) by different cells and tissues in pathological contexts such as tumors, grafted organs, pathogen-infected cells, and inflammatory tissues. HLA-G interacts with specific receptors on T and B lymphocytes, NK cells, neutrophils, and antigen presenting cells, inhibiting their function. HLA-E is virtually expressed by all nucleated cells and binds peptides derived from HLA-class I molecules leader sequence. In physiological conditions, it interacts with CD94/NKG2A inhibitory receptor on NK cells, inhibiting their cytotoxicity against cells displaying normal HLA-class I molecules expression. When such molecules are downregulated (i.e., transformed or infected cells), HLA-class I-derived peptides are lower and subsequently HLA-E expression is dampened, allowing NK cells lo lyse these cells. However, different transformed cells upregulate HLA-E expression to avoid NK-cell mediated lysis. Limited information is available regarding HLA-F function. This molecule acts as chaperone for the *β*2-microglobulin-free heavy chain of other HLA-class I molecules, and it is expressed on the surface of activated lymphocytes. So far, no functional HLA-H molecules encoded by HLA-H genes have been characterized.

In the present issue dedicated to these nonclassical HLA-class Ib molecules, two papers have analyzed some basic aspects of HLA-G. First, E. C. Castelli et al. have reviewed some interesting data regarding the regulation of HLA-G gene expression. They analyzed different mechanisms that operate at transcriptional and posttranscriptional level, with emphasis on polymorphisms that are present both in the untranslated and coding region. Second, E. Alegre et al. have described the factors involved in the generation of different HLA-G isoforms. Moreover, the authors analyzed the data present in the literature regarding posttranslational modifications of HLA-G molecules and the expression and function of different HLA-G specific receptors.

The murine homologue of HLA-G and –E and Qa2 and Qa1 has been analyzed by B. L. Melo-Lima et al., who have provided some interesting data regarding the expression of these molecules during fetal and postnatal development of thymus and other tissues.

Three papers have explored the role of HLA-G in physiological conditions. The review by M. Dahl et al. has analyzed factors that regulate HLA-G expression during pregnancy, with emphasis on differential spatiotemporal expression of HLA-G. Moreover, the authors analyzed functional differences between membrane-bound and soluble HLA-G. F. Montespan et al. have demonstrated that human mesenchymal stem cells derived from bone marrow or adipose tissue maintain HLA-G expression and consequently retain their immunoregulatory properties after osteodifferentiation, thus suggesting that these cells may be used for bone repair. Finally, E. Alegre et al. have analyzed HLA-G biochemistry with special emphasis to the mechanisms that regulate its expression and how the protein modifications affect the quantification.

The role of HLA-G in pathological conditions is the key topic of this special issue. M. Ezeakile et al. have demonstrated that the presence of HLA-G dimers in kidney transplant patients prolonged kidney allograft survival. L. Amiot et al. have analyzed data regarding HLA-G expression in patients with bacterial, viral, or parasitic infections, with particular attention on factors released by parasites that may affect HLA-G expression. Novel interesting data regarding HLA-G expression and function in human tumors have been described in two papers. G. Locafaro et al. have demonstrated that HLA-G expressing DC-10 and CD4^+^ T cells are highly represented in AML patients with HLA-G^+^ blasts and may contribute to immune escape of tumor cells. In contrast, M. J. Rutten et al. have demonstrated that HLA-G expression on tumor cells in ovarian carcinoma patients is an independent prognostic factor that predicts a better overall and event-free survival and response to chemotherapy.

Finally, the expression and function of HLA-E have been investigated by F. Morandi et al., whohave demonstrated that IL-27 treatment upregulated HLA-E expression on human monocytes, rendering the latter cells immunosuppressive, through the inhibition of IFN-*γ* secretion by NK cells.

In conclusion, the papers in this issue will help to enrich the current knowledge regarding HLA-Ib molecules and their role in physiological and pathological conditions.

*Fabio Morandi* *Fabio Morandi* *Enrico Fainardi* *Enrico Fainardi* *Roberta Rizzo* *Roberta Rizzo* *Nathalie Rouas-Freiss* *Nathalie Rouas-Freiss*

